# Deamination-independent restriction of LINE-1 retrotransposition by APOBEC3H

**DOI:** 10.1038/s41598-017-11344-4

**Published:** 2017-09-07

**Authors:** Yuqing Feng, Mariam H. Goubran, Tyson B. Follack, Linda Chelico

**Affiliations:** Department of Microbiology & Immunology, University of Saskatchewan, Saskatoon, Saskatchewan S7N 5E5 Canada

## Abstract

The APOBEC3 family of cytosine deaminase enzymes are able to restrict replication of retroelements, such as LINE-1. However, each of the seven APOBEC3 enzymes have been reported to act differentially to prevent LINE-1 retrotransposition and the mechanisms of APOBEC3-mediated LINE-1 inhibition has not been well understood. The prevailing view for many years was that APOBEC3-mediated LINE-1 inhibition was deamination-independent and relied on APOBEC3s blocking the LINE-1 reverse transcriptase DNA polymerization or transport of the LINE-1 RNA into the nucleus. However, recently it was shown that APOBEC3A can deaminate cytosine, to form uracil, on transiently exposed single-stranded LINE-1 cDNA and this leads to LINE-1 cDNA degradation. In this study, we confirmed that APOBEC3A is a potent deamination-dependent inhibitor of LINE-1 retrotransposition, but show that in contrast, A3H haplotype II and haplotype V restrict LINE-1 activity using a deamination-independent mechanism. Our study supports the model that different APOBEC3 proteins have evolved to inhibit LINE-1 retrotransposition through distinct mechanisms.

## Introduction

Transposable elements are DNA sequences that can move from one location of the genome to another. Approximately 45% of the human genome is recognized as being derived from transposable elements^1, 2^. Transposable elements can be grouped into two major classes based on whether they mobilize via a DNA intermediate (i.e., DNA transposons) or an RNA intermediate (i.e., retrotransposons)^1, 2^. DNA transposons, although not being able to mobilize in the current human genome, have played a major role during the evolution of the eukaryotic genome^3–6^. Retrotransposons transpose through mRNA intermediates followed by reverse transcription, synthesis of double-stranded (ds) DNA, and integration at new genomic locations. Retrotransposons can be divided into two classes: Long Terminal Repeat (LTR) retrotransposons (also known as endogenous retroviruses) and the non-LTR retrotransposons. The replication strategy of endogenous retroviruses resembles those of retroviruses transmitted person to person, except that they remain in one host and are transmitted vertically^2^. The present day endogenous retroviruses have acquired so many mutations that they cannot produce infectious virus^2^.

Non-LTR retrotransposons are found throughout the eukaryotes, and examples of non-LTR retrotransposons include LINE-1 (Long Interspersed Nuclear Element-1, L1) and SINE (Short Interspersed Nuclear Element). Human L1s account for ~17% of the genomic DNA with ~500,000 copies identified, and they represent the only autonomous transposable elements that are currently active in humans^7, 8^. Although the vast majority of L1s are inactive due to accumulated mutations or 5′ truncations, the human genome still contains ~80–100 copies of intact L1s that are retrotransposition competent^1, 2, 9, 10^. Full-length L1 contains a 5′ UTR, two Open Reading Frames (ORFs) that are separated by a 63 nt inter-ORF region, and a 3′ UTR that ends with a polyadenosine-rich sequence (poly(A) tail)^11^. Proteins encoded by ORF1 and ORF2 are both required for efficient L1 retrotransposition. ORF1 encodes a nucleic acid binding protein with demonstrated nucleic acid chaperone activity; ORF2 encodes proteins with endonuclease (EN) activities and reverse transcriptase (RT) activities^12–14^. L1-encoded EN preferentially cleaves ssDNA at an AT-rich consensus sequence (5′ TTTT/A, where “/” dictates the cleavage site). L1 RT is a DNA/RNA dependent DNA polymerase, but lacks RNaseH activity, a trait that distinguishes itself from the retroviral RTs^15^. As a result, during cDNA synthesis the RNA strand of the resulting L1 RNA/DNA hybrid could be either degraded by a yet unidentified cellular protein or displaced during (+) strand cDNA synthesis^16^. Cellular RNaseH2 cooperates with flap endonuclease 1 (FEN-1) to remove RNA primers during lagging strand DNA synthesis and serves as a good candidate^17^. Unlike retroviruses and LTR retrotransposons which undergo reverse transcription in cytoplasm, L1 RNA is reverse-transcribed in the nucleus^18, 19^. L1 relies on a mechanism termed the Target Primed Reverse Transcription (TPRT) for retrotransposition^20^.

Despite the positive effects of retroelement mobilizations, such as contributing to genome evolution and gene diversity, excessive transposition events need to be suppressed, as several genetic diseases have been associated with L1-mediated insertional mutagenesis in germline and somatic cells^9, 10^. The retroelement suppression mechanisms include DNA methylation^21, 22^, RNA interference^23, 24^, and cellular DNA repair factors^25, 26^. Also, studies suggest that several individual proteins are involved in L1 restriction. Examples of these proteins include: Three primer repair Exonuclease 1 (TREX1)^27^, Zinc finger Antiviral Protein (ZAP)^28^, RNA helicase Mov10^29^, RNaseL^30^, SAM domain and HD domain-containing protein 1(SAMHD1)^31, 32^, and the APOBEC family of cytosine deaminases^33–41^.

The APOBEC enzyme family emerged at the origin of vertebrates and have diversified throughout the vertebrate lineage^42, 43^. The most ancient APOBEC family members include Activation Induced cytidine Deaminase (AID) and APOBEC2 (A2) proteins that are identified in jawless and cartilaginous fish^43, 44^. AID initiates class-switch recombination and somatic hypermutation, two processes that occur during antibody maturation, by deaminating cytosine in single-stranded (ss)DNA to form promutagenic uracil. The ssDNA is available during transcription and initiates an error-prone DNA repair process that diversifies the antibody genes and enables a specialized recombination to occur for immunoglobulin class switching^45^. The A2 enzyme is involved in cardiac and skeletal muscle development^46, 47^. APOBEC4 (A4) and APOBEC5 (A5) then emerged in amphibians, although their functions are yet to be identified^48, 49^. During tetrapod evolution, duplication of AID led to the emergence of APOBEC1 (A1)^3, 48, 50^. A1 plays an important role in lipid transport by editing the apolipoprotein B mRNA in intestinal cells by introducing a uracil that results in a stop codon and truncated version of Apolipoprotein B that has a different function than the full length form^51^. A1 can also deaminate cytosine in ssDNA^52^. In placental mammals the APOBEC3 (A3) locus evolved and greatly expanded, which is thought to partly be due to the increasing number of retrotransposons in the genome^43^. While only one *A3* gene is present in mice, up to seven *A3* genes (*A3A, A3B, A3C, A3D, A3F, A3G, A3H*) are present in primates^43^. *A3* genes have likely acquired the anti-L1 activities from the *Activation Induced Cytidine Deaminase (AICDA)/A2*-like gene from which they have evolved. Several studies have demonstrated that in a cell culture system that A3 family members could inhibit a number of mammalian L1s^33–41^. Since *A3* genes are only limited to mammalian lineages, but L1s are far more ancient, dating back to the emergence of vertebrates, it was not surprising to find that APOBEC proteins, such as pre-mammalian AID and reptilian A1 from green anole lizard, are indeed capable of inhibiting L1 retrotransposition^36, 53^.

A3s have been reported to restrict L1 by both deamination -dependent or -independent mechanisms^34, 35, 38, 40, 41, 54–57^. For a deamination-dependent mechanism, during the reverse transcription of the L1 RNA, the (-)cDNA gets transiently exposed after RNA template degradation, which renders it susceptible to A3-mediated cytosine to uracil (C → U) deaminations^57^. The uracils present on L1 cDNA could trigger cDNA degradation through the actions of the host base excision repair pathway. Specifically, cellular uracil DNA N-glycosylase (UNG) excises the uracils from ssDNA, generating abasic sites and apurinic/apyrimidinic endonuclease (APE) produces nicks on the DNA which would induce L1 cDNA degradation. Alternatively, a uracil-containing cDNA may escape the degradation, become replicated to form a dsDNA, and integrate into the host chromosome, but be functionally inactivated through A3 induced C/G → T/A transition mutations that result from the polymerase using uracil as a template. For a deamination-independent mechanism of L1 restriction, the A3s can sequester L1 ribonucleoprotein (RNP) complexes to cytoplasmic compartments such as stress granules or physically interact with L1 RT and inhibit DNA polymerization during TPRT^41, 58, 59^. There is no clear agreement in the literature as to whether A3s utilize the deamination -dependent or -independent mode for L1 restriction. These ambiguities arose because no G → A mutations on the coding strand, which would indicate the occurrence of C → U deamination on the L1 cDNA, could be detected. However, none of these cell culture studies blocked the action of UNG^34–36, 60^. Richardson *et al*., reasoned that the unsuccessful attempts to uncover A3-induced mutations were likely due to the degradation of uracil-containing cDNA intermediates by the UNG and APE-mediated DNA repair proteins^57^. In their study, using the Uracil Glycosylase Inhibitor (UGI) protein of *Bacillus subtilis* bacteriophage PBS1^61^, they demonstrated that A3A restricted L1 through a deamination-dependent mechanism, suggesting that the mechanism by which A3 enzymes restricted L1 needed to be readdressed. These data also explained how mammalian L1 sequences analyzed on larger genome-wide scales could show more evidence of cytosine deamination and is in agreement with the known restriction mechanism of several endogenous retroviruses that show a G → A mutation bias^62–65^.

To address whether the deamination-dependent mechanism of L1 inhibition is unique to A3A or common to other A3s, we examined how A3H restricted L1 retrotransposition in cell culture. There are at least seven A3H haplotypes that exist in humans. Three of the haplotypes are stable and catalytically active (II, V, VII), three are thermodynamically unstable and have no discernable activity (III, IV, VI), and one has an intermediate stability and is catalytically active (I)^66–70^. Since haplotype II (hap II) and haplotype V (hap V) comprise the majority of the stable haplotypes we examined how they restricted L1 retrotransposition. Previous research has demonstrated that stable A3H could inhibit L1, however, a mechanism was not identified^67, 71^. In this study, we demonstrate that both A3H hap II and hap V suppress L1 to a similar level but the inhibition of L1 does not involve DNA deamination, in contrast to A3A.

## Results

To establish a context with which to interpret our results with A3H, we also included A3A and A3G in our study. These additional A3s were chosen based on previous publications indicating that A3A was a strong inhibitor of L1 retrotransposition and A3G was unable or weakly able to inhibit of L1 retrotransposition^34, 39, 40, 54^.

### Effect of A3A, A3G, and A3H on L1 retrotransposition

**Figure 1:**
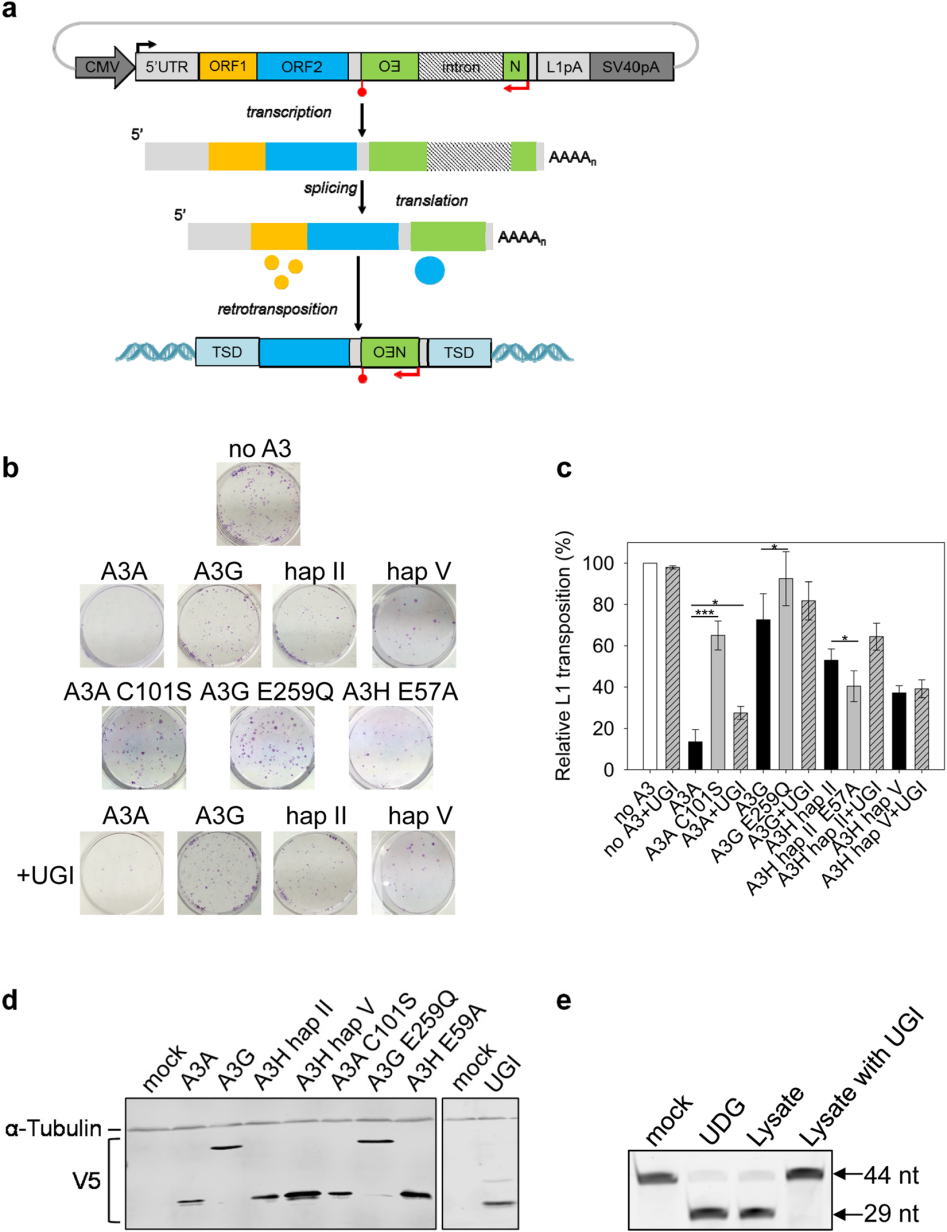
Restriction of L1 retrotransposition by A3A, A3H, and A3G. (**a**) Schematic for L1 retrotransposition assay. A full-length retrotransposition-competent L1 (~6 Kb) that contains a neo-reporter cassette (green box) in the 3′UTR region was used. The reporter gene, *neomycin phosphotransferase* (backwards NEO), is in the opposite transcription orientation with respect to L1. Only upon reverse transcription and integration into a genomic locus can the *neo* gene be expressed to confer resistance to G418. The retrotransposed L1 is usually 5′ truncated and integration sites are usually flanked by target site duplications (TSD) sequences. (**b**) Representative experimental results of the *neo*-based L1 retrotransposition assay. (**c**) Quantification of the effects of various A3s on L1 transposition efficiencies in the presence or absence of UGI. Results from experiments in the absence of UGI are normalized to L1 with empty vector (no A3). Results from experiments in the presence of UGI are normalized to L1 + UGI. For comparison, on the graph the L1 + UGI is normalized to no A3 to demonstrate that UGI alone does not increase the restriction of L1. Results are the average of at least three independent experiments. Error bars represent the standard deviation. Designations for significant difference of values determined by a T-test were p ≤ 0.001 (***), p ≤ 0.01(**), or p ≤ 0.05 (*). (**d**) The cellular expression of V5-tagged A3 enzymes and UGI in HeLa cells was confirmed by immunoblotting using anti-V5 antibodies. The α-tubulin expression was used as a loading control and detected in parallel with the V5 tag. Detection of V5 for A3 enzymes and UGI was done on two separate blots. The UGI blot was cropped from a blot containing lanes with additional experimental replicates for conciseness. (**e**) A cell lysate based assay determined that UGI-V5 expression in HeLa cells inhibited endogenous UNG. From left to right, the lanes show the 44 nt deoxyuridine containing ssDNA oligonucleotide (mock), the ssDNA with commercially available UDG which generates a 29 nt band after uracil removal and heating of ssDNA under alkaline conditions, the ssDNA with HeLa cell lysate, and the ssDNA with HeLa cell lysate from cells that were transiently transfected with UGI.

To study the influence of A3 proteins on L1 retrotransposition, a pre-established cell line based retrotransposition assay was performed^72, 73^ (Fig. 1a). To detect retrotransposition events, the 3′ untranslated regions (3′UTRs) of a human L1 retrotransposon vector (pJM101/L1.3) is marked with a *neomycin phosphotransferase (neo)* reporter cassette, which, when expressed, can convey resistance to G418. The *neo* reporter cassette has its own promoter and polyadenyation signal, and it consists of an antisense *neo* gene disrupted by an intron of the *γ-globin* gene, which is in the same transcriptional orientation as the L1 element (Fig. 1a). Because the *neo* gene is rendered inactive by the presence of an intron, G418-resistant (G418^R^) cells will only arise when a transcript initiated from the L1 promoter is spliced, reverse transcribed, its cDNA is re-inserted into the host chromosome, and the *neo* gene is transcribed from an internal promoter (Fig. 1a). The L1 assay relies on *neo* expression as a readout and the rise of a G418^R^ colony demonstrates a successful L1 retrotransposition event. Hela cells were utilized in this assay, as these cells contain a low endogenous L1 copy number (~25 copies per cell)^38^, can accommodate high levels of L1 retrotransposition events from ectopically expressed L1 reporter plasmids^73, 74^ and do not express endogenous A3A, A3G and A3H proteins^33, 38^.

In order to study the effect of A3 proteins on L1 retrotransposition, HeLa cells were co-transfected with the L1 reporter plasmid pJM101/L1.3 in the presence and absence of plasmids expressing V5-tagged A3s. Three days post transfection, the cells were subjected to G418 selection for 11 days and the G418^R^ colonies were stained and counted. Transfection of L1 vector alone with no A3 expression plasmid generates a quantifiable amount of G418^R^ colonies (Fig. 1b, no A3). In the presence of A3A and A3G, L1 retrotransposition efficiency decreased to 13.5% and 72.6%, respectively (Fig. 1b,c). These data are in agreement with previous studies that demonstrated A3A potently inhibits and A3G does not have a significant inhibitory effect on L1 retrotransposition in cultured cells^34, 38, 40, 54^. A3H hap II and hap V decreased the L1 retrotransposition efficiency to 53.0% and 37.2%, respectively, demonstrating that the two A3H haplotypes can restrict L1 retrotransposition, but their effect is 3- to 4 -fold less than A3A (Fig. 1b,c).

### A3H restricts L1 by a deamination-independent mechanism

We investigated whether the inhibition of L1 by A3H involved deamination by creating a catalytic mutant, E57A that prevents coordination of water with the Zn^2+^ molecule in the active site^75, 76^. Similar mutants were made in A3A (C102S, disruption of Zn^2+^ coordination) and A3G (E259Q, disruption of water coordination) according to the previously published catalytic mutants^34, 77, 78^. In this experiment we tested only A3H hap II since both hap II and hap V restricted L1 retrotransposition similarly (Fig. 1c). Consistent with previous data, the efficient inhibition of L1 by A3A required deaminase activity, since the retrotransposition frequency in the presence of A3A C102S was 65.0%, a 4.8-fold increase in retrotransposition efficiency from the wild type A3A condition (Fig. 1b,c)^57^. The L1 retrotransposition efficiency in the presence of A3G E259Q was similar to wild type A3G, and although a statistically significant difference was found, it was only a 1.3-fold change, further confirming that A3G only imposes a minimal effect on L1 (Fig. 1b,c, retrotransposition efficiencies of 72.6% and 92.4%). Similarly, the A3H hap II E57A mutant had a significant, although only 1.3-fold, difference from wild type A3H hap II in restricting L1 retrotransposition, providing evidence that deaminase activity is not required for A3H-mediated L1 restriction (Fig. 1b,c, 53.0% for hap II and 40% for A3H hap II E57A). To exclude the possibility that the observed differences in retrotransposition efficiencies were due to different cellular A3 levels, we used immunoblotting to detect the A3 expression levels in the HeLa cells (Fig. 1d). The wild type and catalytic mutant A3 enzymes were expressed at equivalent levels (Fig. 1d). As a result, these data further confirm that A3A restriction of L1 is deamination-dependent and A3H restriction of L1 is deamination-dependent.

To extend these results we also cotransfected an expression plasmid for UGI into the HeLa cells during the experiment. The UGI is a small 9.5 kDa protein that inhibits UDG/UNG from bacterial and human cells by reversible protein binding at a 1:1 stoichiometry^79, 80^. The UGI-V5 expressed well in the HeLa cells (Fig. 1d). We also tested the UNG activity by lysing HeLa cells that were or were not transfected with UGI and incubating them with a 44 nt single-stranded DNA oligonucleotide containing a single deoxyuridine (Fig. 1e). The endogenous UNG in the cell lysates was able to completely excise the uracil from the ssDNA and the UGI was able to completely inhibit this process (Fig. 1e). Consistent with a previous study, the inhibition of L1 retrotransposition by A3A was reduced 2-fold when UGI was expressed (Fig. 1b,c, from 13.5% to 27.5%), indicating the L1 inhibition imposed by A3A indeed involves deamination^57^. Since the UGI was able to completely inhibit the UNG in cell lysates (Fig. 1e), the transient transfection of the UGI likely resulted in incomplete inhibition of UNG due to not every transfected cell receiving all three expression vectors (Fig. 1b,c), which is why we could not recover higher amounts of retrotransposition^57^. In the presence of A3G, the UGI expression did not cause a significant increase in L1 retrotransposition efficiency (Fig. 1b,c). For A3G, the retrotransposition efficiency in the presence and absence of UGI was 82.8% and 72.6%, respectively (Fig. 1c). For A3H hap II and hap V, the retrotransposition efficiencies in the presence and absence of UGI were also similar (Fig. 1b,c, A3H hap II, 64.4% and 53.0%; A3H hap V, 39.2% and 37.2%).

### A3-induced mutagenesis of transposed *neo* gene

**Figure 2:**
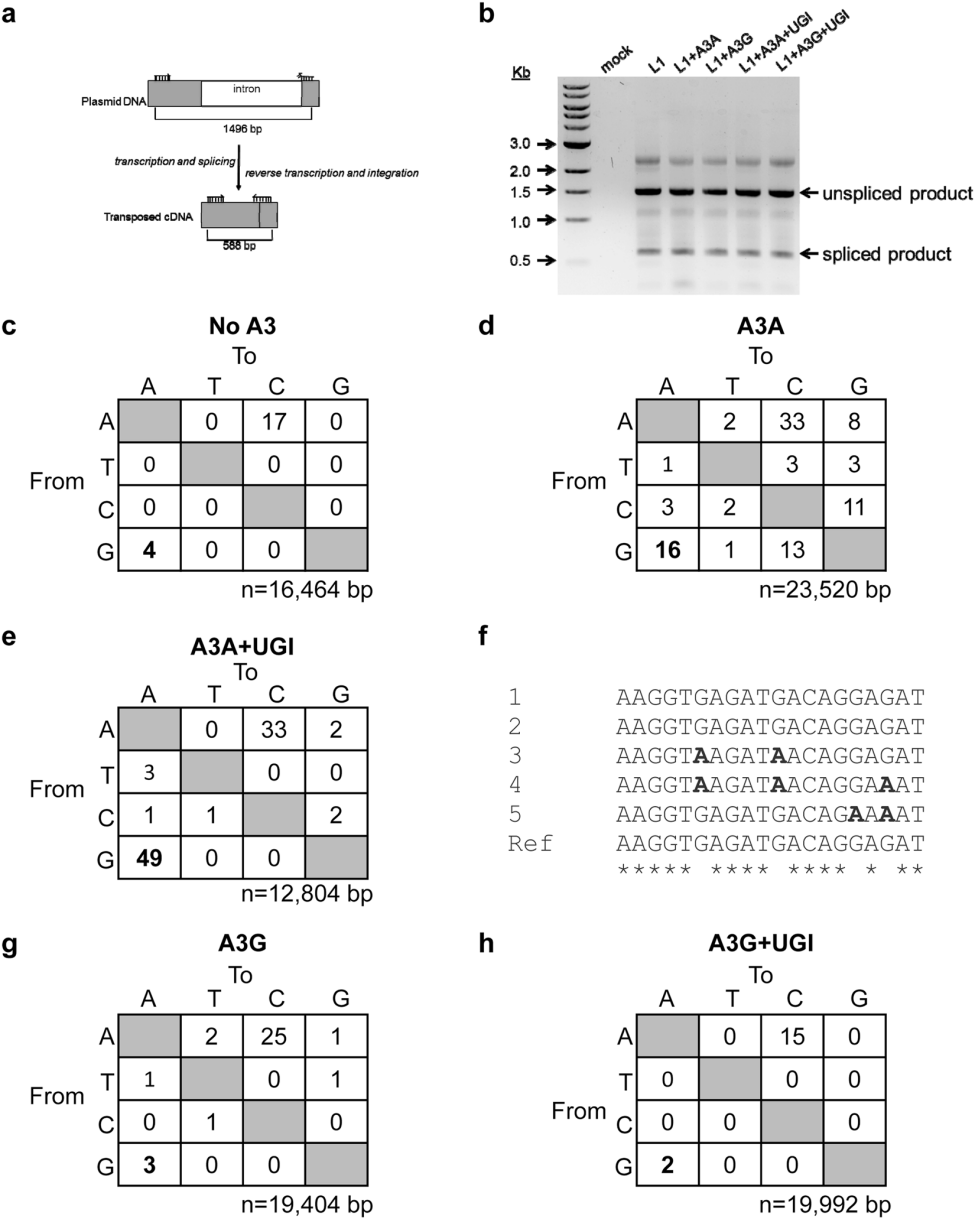
A3A, but not A3G, promotes G→A mutations in the transposed *neo* gene. (**a**) Schematic of the *neo*-cassette before and after L1 retrotransposition with specific primers and the size of the expected PCR fragments indicated. (**b**) A representative agarose gel of the PCR products amplified using the primers shown in (**a**). (**c**) Sequencing of *neo* spliced product from cells where no A3 was transfected demonstrated the background G→A mutation frequency was 0.24 mutations/kb. (**d**) In the presence of A3A, the G→A mutation frequency was 0.68 mutations/kb. (**e**) The UGI expression increased the A3A G→A mutation frequency to 3.8 mutations/kb. (**f**) DNA sequencing data from a subset of clones shows the sequence context of G→A mutations induced by A3A. Asterisks denote homology. (**g**–**f**) A3G G→A mutation frequencies in the (**g**) absence (0.16 mutations/kb) and (**h**) presence (0.10 mutations/kb) of UGI were similar. The value of n is the total number of nucleotides analyzed.

**Figure 3:**
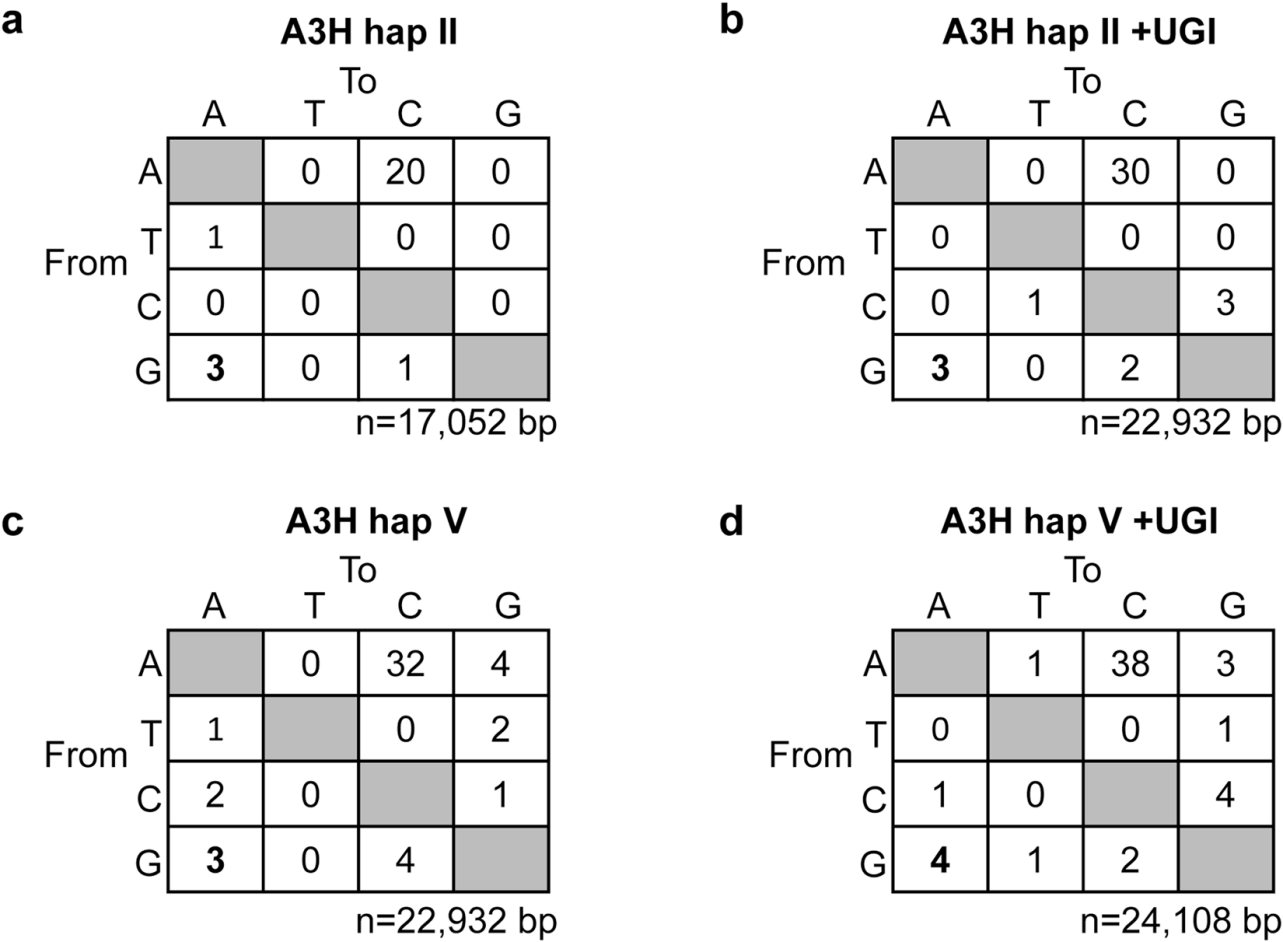
A3H hap II and hap V do not induce G→A mutations in the transposed *neo* gene. (**a**,**b**) In the (**a**) absence (0.18 mutations/kb) and (b) presence (0.13 mutations/kb) of UGI, A3H hap II induced a similar G→A mutation frequency. (**c**,**d**) Similarly, for A3H hap V, there was no difference in the G→A mutation frequency in the (**c**) absence (0.13 mutations/kb) or (**d**) presence (0.16 mutations/kb) of UGI. The value of n is the total number of nucleotides analyzed.

To determine if A3-mediated deaminations in transposed L1 were occurring we amplified a segment of the L1 construct for DNA sequencing. The detection of L1 retrotransposition events in the assay relies on the expression of the spliced *neo* gene. However, the neo-cassette is located within the 3′ UTR and would remain single-stranded for the longest time during reverse transcription since it is furthest from the priming site for the second DNA strand, which would make this region most vulnerable to mutagenesis. To avoid mutated clones being lost in the G418-resistance selection process, we extracted total cellular DNA three days post-transfection and prior to G418 selections. Because an intron was inserted into the *neo* gene and a spliced *neo* gene could be produced only after an L1 integration event (Fig. 1a), we designed primers that annealed to exon regions spanning the intron, enabling amplification of PCR products with different lengths that correspond to the spliced and unspliced products (Fig. 2a). The UGI expression plasmid was also transfected during some of these experiments to promote the recovery of mutations. Several bands were observed in the PCR amplification products even with the primers that were designed to eliminate the amplification of intron sequences (Fig. 2b), in agreement with the previous observation that retrotransposition is a rare event^34^. Sequencing confirmed the 588 bp species corresponded to the spliced, transposed *neo* products, whereas the 1496 bp species corresponded to the PCR amplification of the unspliced, full-length *neo* from the plasmid DNA. Interestingly, during sequence analysis of the spliced *neo* gene we noticed a high percentage of A → C transversion mutations (Figs 2 and 3). These A → C mutations were persistently observed across different transfection conditions, suggesting that this may be an insertion bias of the L1 reverse transcriptase (Fig. 2 and Fig. 3). Even in the absence of A3 enzymes, G → A transition mutations accounted for the second most frequent mutation type (Fig. 2c, no A3, 0.24 mutations/kb).

In the presence of A3A, the G → A mutation rate increased ~3-fold to 0.68 mutations/kb (Fig. 2d). When UGI was co-transfected along with L1 and A3A, a ~6-fold increase in G → A mutation rate was observed from A3A alone (Fig. 2e, A3A + UGI, the G → A mutation rate is 3.8 mutations/kb). This suggests that introducing UGI blocked UNG and allowed more G → A mutations to be uncovered, supporting that restriction of L1 retrotransposition is at least in part due to degradation of uracil-containing L1 intermediates. However, since A3A-induced G → A mutations were recovered in the absence of UGI, some L1 intermediates can still escape the degradation resulting from DNA repair enzymes and integrate into the host chromosome. Consistent with A3A ssDNA cytosine deamination activity, very few C → T mutations were observed on the transposed L1 coding strands, excluding the possibility of A3A deaminating L1 mRNA (Fig. 2d,e). Further, sequencing of the unspliced *neo* gene did not recover any mutations confirming that A3A is not deaminating the transfected plasmid DNA before mRNA splicing (data not shown), although this has been observed under other experimental conditions^81^, and that indeed the mutations were acquired during integration of the L1 by TPRT, consistent with Richardson *et al*.^57^. Because A3A prefers to deaminate C → U within a 5′TC or 5′CC context (underlined C is deaminated)^82, 83^, we determined whether these G → A mutations on the genomic strand occurred within the preferred deamination motif as a confirmation of A3A deamination activity. Deamination of these motifs in the cDNA will result in 5′GA → 5′AA or 5′GG → AG mutations in the coding strand. Out of the 16 G → A mutations we recovered in the *neo* gene, two mutations were within the 5′GA → 5′AA context and 1 mutation was within the 5′GG → AG context (data not shown). In addition, other mutations at 5′GC → 5′AC and 5′GT → 5′AT motifs were observed that may or may not be due to A3A catalytic activity (data not shown). However, in the presence of UGI, the majority of the G → A mutations occurred within the 5′GA → 5′AA context, confirming that UGI blocked removal of uracils formed by A3A catalytic activity (Fig. 2f). Consistent with the previous observation that A3G has little inhibitory effect on L1 activity (Fig. 1b,c), a G → A mutation frequency similar to that of the background, was observed in *neo* gene in the presence and absence of UGI (Fig. 2g,h, G → A mutation frequencies of 0.16 and 0.10 mutations/kb). These data suggest that any minor inhibitory effect of L1 retrotransposition by A3G (Fig. 1c, 72.6%) was not caused by A3G-mediated deamination. Taken together, these data strengthen the findings that A3A inhibits L1 retrotransposition using a deamination-dependent mode, whereas A3G does not seem to affect L1 retrotransposition.

For A3H, we expected that similar levels of G → A mutations would be observed regardless of the presence of UGI since the A3H hap II wild type and E57A restricted L1 retrotransposition similarly (Fig. 1c). Consistent with transposon efficiency data, no enhanced rates of G → A mutations were detected in the *neo* gene in the presence of A3H hap II (Fig. 3a,b, mutation frequencies of 0.18 mutations/kb and 0.13 mutations/kb). Similar results were found for A3H hap V (Fig. 3c,d). Furthermore, none of the G → A mutations occurred within the preferred 5′GA → 5′AA context, which represents A3H deamination at 5′TC sites (data not shown)^66^. A3H hap II and hap V also did not appear to be able to deaminate L1 mRNA, since no C → T mutations with the correct sequence context were found (Fig. 3).

## Discussion

In this study, we showed that different A3 family members have varying abilities and mechanisms to inhibit the replication of the retrotransposon L1. Specifically, A3A acts as a potent inhibitor for L1 retrotransposition using primarily a deamination-dependent mechanism, A3G does not appear to inhibit L1, and A3H does inhibit L1, consistent with previous studies^34, 35, 38, 39, 54, 60^. Unique to our study is that we determined A3H hap II and hap V inhibit L1 using a deamination-independent mechanism.

Numerous studies have reported the inhibitory effects of different A3 family members on L1, and in general a deamination-independent L1 restriction model was proposed due to an inability to detect G → A mutations^34, 35, 38–40, 54–56^. Richardson *et al*., studied the effects of A3A on L1 retrotransposition in a cell line that stably expressed UGI, and this allowed the recovery of A3A-mediated deamination events in the retrotransposed L1 sequences^57^. However, our study shows that deamination-dependent L1 restriction is not a common property for all A3 enzymes, since A3H hap II and hap V did not induce G → A mutations, even in the presence of UGI (Fig. 3).

A3A has been demonstrated to be the most potent inhibitor of L1 replication and can restrict L1 retrotransposition frequencies by 75% to 95%, depending on the transfection conditions^34, 38, 40, 54, 57^. In our system A3A decreased L1 retrotransposition frequency by 86.5% and the catalytic mutant, A3A C101S, could only decrease L1 retrotransposition frequency by 35.0% (Fig. 1b,c), indicating that the deamination-dependent mechanism of A3A contributed the majority of the L1 restriction activity. This is consistent with DNA sequencing where we found that UGI expression alleviated A3A-mediated L1 inhibition by 2-fold (Fig. 1b,c, from 13.5% to 27.5%). These data are also in agreement with Richardson *et al*.^57^.

We found A3H hap II and hap V are able to suppress L1 retrotransposition to a moderate level and there were no apparent differences in their L1 restriction abilities (Fig. 1b,c). Three lines of evidence suggest both A3H haplotypes inhibit L1 mobilization in a manner that does not involve deamination. First, the L1 retrotransposition efficiency was the same in the absence or presence of UGI when either A3H hap II or hap V were expressed (Fig. 1b,c). Second, we found that hap II and hap V produced a mutation profile with G → A mutations at the background level either in the absence or presence of UGI (Figs 2c and 3). Third, the A3H hap II catalytic mutant E57A decreased L1 retrotransposition to an equivalent degree as the wild type A3H hap II (Fig. 1b,c). Although there is no prerequisite for a specific cellular localization for an A3 to restrict L1, there does appear to be different mechanism^38^. For A3A that localizes to the nucleus when ectopically expressed, a deamination-dependent mechanism is involved^84^ (Fig. 1b,c). However, for A3H hap II and V that are primarily cytoplasmic, a deamination-independent mechanism is involved^85^ (Fig. 1b,c). Cytoplasmic A3s can inhibit L1 by binding ORF1 in an RNA-dependent manner and preventing the nuclear import of the L1 RNP complex^41^. In contrast, A3s that locate in nucleus have been reported to inhibit L1’s TPRT process either by deaminating (-)cDNA or physically impair RT’s activity^56, 57, 86^. Based on these observations and that both A3H Hap II and Hap V are primarily cytoplasmic proteins^67^, we hypothesize A3H could inhibit L1 mobility by binding to the L1 RNP in the cytoplasm, similar to what has been reported for A3C and A3D^41, 59^.

The analysis of the L1 restriction ability of A3A, A3G, and A3H hap II and hap V have demonstrated that each of the A3s has a unique effect on L1. In particular, although L1 restriction by A3s can be deamination-dependent, as shown for A3A, this does not appear to be applicable to all A3 enzymes. These data suggest that reanalysis of A3-mediated L1 restriction in the presence of UGI for all A3 family members is warranted to fully understand their mechanism of activity.

## Materials and Methods

### Plasmids, cloning and site-directed mutagenesis

L1 expression plasmid pJM101/L1.3-Neo was kindly provided by Dr. John V. Moran (University of Michigan). The cDNA of A3A, A3G and A3H have been previously reported^82, 87, 88^ and were cloned into pcDNA6-V5/His.A using XbaI and XhoI cloning sites giving the enzymes a C-terminal V5-His fusion tag. Mutants of A3 enzymes (A3A C101S, A3G E259Q, A3H E57A) were made using the wild type construct for site-directed mutagenesis (QuickChange site-directed mutagenesis protocol, Agilent). The Uracil DNA glycosylase inhibitor gene was synthesized by GenScript with codon optimization for human cells and subcloned into pcDNA6, but was expressed without the tags for the experiments or subcloned into pcDNA3.1 with a C-terminal V5 tag for checking expression by immunoblotting. All constructed plasmids were verified by sequencing.

### Retrotransposition assay

HeLa cells were seeded in each well of a 6-well tissue culture plate at a density of 2 × 10^5^ cells/well in Dulbecco’s Modified Eagle Medium (DMEM) with 10% FBS. Approximately 22 h after plating, Hela cells were co-transfected with 1 μg of L1 retrotransposon encoding plasmid (pJM101/L1.3) and 1 μg of an A3 encoding plasmid or empty vector. Transfections were performed using GeneJuice (EMD Millipore) according to the manufacturer’s protocol. Media was replaced the following day. Approximately 48 h post-transfection, cells were re-seeded into 6-well plates (at 1 in 5 dilution) and the next day attached cells were subjected to selection with 400 μg/mL G418 (Life Technologies). During the reseeding process, cells were checked for viability by trypan blue staining. We consistently observed a cell viability > 95% indicating that expression of A3A, A3G, or A3H did not cause cellular toxicity. G418 selection was carried out for 11 days and the selection media were replaced every other day. Afterwards, G418^R^ colonies were washed twice with PBS, fixed with 2% paraformaldehyde/0.4% glutaraldehyde, and stained with 0.1% crystal violet solution.

### Immunoblotting

To detect A3 expression levels, cells were washed in 1x PBS, harvested in Laemmli sample buffer (58 mM Tris (pH 6.8), 5% glycerol, 2% sodium dodecyl sulphate (SDS), 1.5% dithiothreitol (DTT)), and 40 μg of total protein was resolved by SDS-PAGE. Proteins were transferred to a nitrocellulose membrane and A3 or UGI in the cell lysates were probed with primary antibodies (mouse monoclonal V5, 1:1000 (Sigma) and rabbit monoclonal α-tubulin, 1:1000 (Sigma)). After incubation with the secondary antibodies (A3 proteins: IRDye 800-labeled goat anti-mouse; α-tubulin: IRDye 680-labeled goat anti-rabbit) the tagged proteins and loading control was detected simultaneously on the same membrane by using the Licor/Odyssey system.

### Nucleic acid extraction and PCR

Hela cells were co-transfected with 1 μg of A3 and L1 plasmids in the presence or absence of 1 μg UGI vector as described above. Cellular DNA from the transfected cells was extracted 72 hr post-transfection using DNAzol reagent (Thermo Fisher). Extracted DNA was used as a template for PCR amplification of the spliced *neo* gene using the forward primer (G418Neoseq For) 5′-TCGGGAGCGGCGATACCGT-3′ and the reverse primer (G418Neoseq Rev) 5′-CGGTGCCCTGAATGAGCTTCA-3′ with the Q5 High-Fidelity polymerase (New England Biolabs). The PCR cycle used had an initial denaturation step at 98 °C (3 min) followed by 35 cycles of amplification (30 sec at 98 °C, 30 sec at 65.5 °C and 18 sec at 72 °C). PCR products were electrophoresed to separate unspliced (of plasmid origin) and spliced (of integrated L1 origin) PCR products. The spliced product was gel purified with the GenElute Gel Extraction kit (Sigma) and cloned into pJET1.2/blunt vector (CloneJet PCR cloning kit, Thermo Scientific). After transformation into *Escherichia coli* DH5α cells, clones were picked and sent for sequencing using a kit-specific primer (pJET1.2 Forward) at the National Research Council DNA sequencing Facility (Saskatoon, SK). Sequence alignments were performed using Clustal Omega.

### Uracil DNA Glycosylase Inhibitor assay

HeLa cells were transfected with 1 μg of L1 retrotransposon encoding plasmid (pJM101/L1.3) and either 1 μg empty vector or UGI vector as described above. Cells were harvested and lysed 72 h after the transfection in buffer containing 50 mM Tris-Cl pH 7.4, 1% Nonidet-P40, 0.1% sodium deoxycholate, 10% glycerol, 150 mM NaCl, 1 mM DTT, 20 μg/mL RNaseA and EDTA-free protease inhibitor (Roche). To test for UGI activity, 8 μL of the lysate was incubated with 100 nM of a 44 nt ssDNA oligonucleotide with a single deoxyuridine and a 5′ Fluorescein (5′ (Fluorescein)-AAA GAG AAA GTG ATA AAC AAA GAG TAA AGU AGA TAG AGA GTG ATA 3′) for 15 min at 37 °C. To cleave the DNA at abasic sites generated by the action of UNG, the DNA was then heated at 95 °C for 10 min in the presence of 0.2 M NaOH. Purified UDG from *E. coli* (New England Biolabs) was used as a control. For DNA visualization the samples were mixed with an equal volume of formamide/EDTA loading dye, resolved on a urea denaturing 16% v/v polyacrylamide gel, and scanned using a Typhoon Trio multipurpose scanner (GE Healthcare).

### Data Availability

No datasets were generated or analyzed during the current study.
